# Wearable Sensing System for NonInvasive Monitoring of Intracranial BioFluid Shifts in Aerospace Applications

**DOI:** 10.3390/s23020985

**Published:** 2023-01-14

**Authors:** Jacob L. Griffith, Kim Cluff, Grant M. Downes, Brandon Eckerman, Subash Bhandari, Benjamin E. Loflin, Ryan Becker, Fayez Alruwaili, Noor Mohammed

**Affiliations:** 1 Department of Biomedical Engineering, Wichita State University, Wichita, KS 67260, USA; 2 J. Crayton Pruitt Family Department of Biomedical Engineering, University of Florida, Gainesville, FL 32611, USA; 3 Pharmaceutical Chemistry, University of Kansas, Lawrence, KS 66045, USA; 4 Meinig School of Biomedical Engineering, Cornell University, Ithaca, NY 14850, USA; 5 Department of Orthopaedic Surgery, Indiana University, Indianapolis, IN 46202, USA; 6 Department of Biomedical Engineering, Duke University, Durham, NC 27708, USA; 7 Department of Biomedical Engineering, Rowan University, Glassboro, NJ 08028, USA; 8 Department of Electrical and Computer Engineering, University of Massachusetts Amherst, Amherst, MA 01003, USA

**Keywords:** electromagnetic sensing, intracranial pressure, microgravity, lower body negative pressure, RF resonator

## Abstract

The alteration of the hydrostatic pressure gradient in the human body has been associated with changes in human physiology, including abnormal blood flow, syncope, and visual impairment. The focus of this study was to evaluate changes in the resonant frequency of a wearable electromagnetic resonant skin patch sensor during simulated physiological changes observed in aerospace applications. Simulated microgravity was induced in eight healthy human participants (n = 8), and the implementation of lower body negative pressure (LBNP) countermeasures was induced in four healthy human participants (n = 4). The average shift in resonant frequency was −13.76 ± 6.49 MHz for simulated microgravity with a shift in intracranial pressure (ICP) of 9.53 ± 1.32 mmHg, and a shift of 8.80 ± 5.2097 MHz for LBNP with a shift in ICP of approximately −5.83 ± 2.76 mmHg. The constructed regression model to explain the variance in shifts in ICP using the shifts in resonant frequency (R^2^ = 0.97) resulted in a root mean square error of 1.24. This work demonstrates a strong correlation between sensor signal response and shifts in ICP. Furthermore, this study establishes a foundation for future work integrating wearable sensors with alert systems and countermeasure recommendations for pilots and astronauts.

## 1. Introduction

The alteration of human biofluid distributions in an aerospace environment has been associated with changes in human physiology, including abnormal blood flow, syncope, and visual impairment [1,2]. On Earth, the gravitational field forms a hydrostatic pressure gradient in the human body, which results in the majority of biofluids being located in the lower body; however, this pressure gradient is disrupted in both microgravity, experienced by astronauts, and hypergravity, experienced by fighter pilots [3]. In microgravity, the modification of the pressure gradient results in a cephalad shift of blood and interstitial fluid (≈1.5–2.0 L) from the lower limbs [3]. In addition to a redistribution of fluids, this shift also upsets the typical cardiovascular regulation of arterial blood pressure [4,5], whereas in a hypergravity environment, a rapid redistribution of fluid away from the head, towards the lower extremities, can occur, causing intracranial hypotension, which may lead to loss of consciousness. The implementation of reliable countermeasures requires an easy-to-use, noninvasive technology capable of detecting and quantifying the magnitude of biofluid shifts, which was the focus of this study. 

Recent investigations have been launched to determine the causes of ophthalmic conditions present in astronauts after long-duration spaceflight [1,6,7,8,9]. These conditions resemble symptoms of elevated intracranial pressure (ICP) on earth, including disc edema, globe flattening, and hyperopic shifts [2,8,10]. The resulting changes in ocular structure and visual function are hypothesized to be associated with the fluid redistribution that occurs upon entering microgravity. As a result, these conditions are referred to as spaceflight-associated neuro-ocular syndrome (SANS) or microgravity ocular syndrome. SANS is considered a health risk by the National Aeronautics and Space Administration (NASA) for long-duration spaceflight [2,10]. Current investigations into the detection and tracking of the redistribution of biofluids in a microgravity environment have focused on using noninvasive, point-of-care technologies to estimate ICP, including ultrasound, otoacoustics, tympanic membrane displacement, and pulse lock loop [11,12,13,14]. However, the lack of a wearable form factor and penetration depth of these technologies present an obstacle in the integration of these technologies to be used by crew members in an aerospace environment. The goal of developing these technologies is to guide the administration of countermeasures so that the physiological effects caused by exposure to a microgravity environment can be mitigated or reversed.

Countermeasures to mitigate the potential risks that astronauts face due to cephalad biofluid shifts are a primary interest in microgravity. A primary countermeasure being investigated to redistribute biofluids in the human body in a microgravity environment is lower body negative pressure (LBNP) [15,16,17]. The implementation and overall effectiveness of LBNP, as well as other countermeasures, rely upon identifying when the countermeasure becomes necessary and upon the duration of LBNP being applied. Through the monitoring of intracranial biofluid levels over time, the proposed sensing system could be utilized to alert crew members when fluid levels increase to a state necessitating the administration of the countermeasure. Conversely, during the administration of the LBNP countermeasure, the real-time monitoring of fluid levels can be used to alert crew members when they have returned to a physiologically healthy status. This monitoring would allow for additional analysis of optimal protocols for applying the LBNP countermeasure.

Our previous investigations with relevance to aerospace applications focused on measuring intracranial volume shifts [18], limb hemodynamics [19,20,21], intraventricular stroke volume [22], physiological temperature [23], and shoulder joint clearance in a space suit [24]. 

Electromagnetic (EM) resonant sensors were the focus of this study, possess the ability to noninvasively detect fluid shifts in preliminary human tests [18] while being lightweight, and can be designed with a wearable form factor similar to an adhesive bandage or wearable headband. EM resonant sensors detect volumetric changes in layered dielectric materials as shifts in the resonant frequency due to changes in the effective permittivity [25,26]. We further outlined the theory and operating principles of EM resonant sensors for biomedical applications in our previous work [18,19,20,21,22,23,24].

The focus of this study was to evaluate changes in the resonant frequency of the EM resonant skin patch sensor as a result of volumetric changes in the cranial cavity during a 15° head-down tilt and during the application of LBNP. We hypothesized that fluid volume changes, as well as subsequent ICP shifts, inside the human body can be noninvasively detected as a shift in the resonant frequency of the EM resonant skin patch sensor. The objectives of this study were to (1) develop a portable, wearable sensor system capable of obtaining measurements that noninvasively detect shifts in intracranial fluid volume, (2) detect changes in the EM sensor’s resonant frequency over time, corresponding to induced shifts in intracranial biofluid volume, and (3) estimate changes in ICP based on the EM sensor’s resonant frequency.

## 2. Materials and Methods

Detecting shifts in ICP requires the ability to detect changes in sensor signal response corresponding with fluid volume changes. To assess the sensor’s ability to detect increases in intracranial fluid volume in a simulated microgravity environment, a head-down tilt test was conducted with the sensing system using 8 healthy male volunteers (mean ± SD (range): age 23.875 ± 2.95 years (21–30); height 179.38 ± 7.30 cm (165–188); and weight 86.75 ± 17.22 kg (66–109)). 

Additionally, the administration of lower body negative pressure (LBNP) protocols was performed using 4 healthy male volunteers (mean ± SD (range): age 22.8 ± 1.79 years (21–25); height 180.85 ± 5.79 cm (173–188); and weight 81.65 ± 17.93 kg (66–104)) to simulate a decrease in intracranial fluid volume. The purpose of the LBNP protocol was to evaluate the sensing technology’s potential to be used to detect a decrease in intracranial biofluid volume, such as the one occurring in fighter pilots undergoing high G-forces.

### 2.1. Sensing System and Data Acquisition

**Figure 1:**
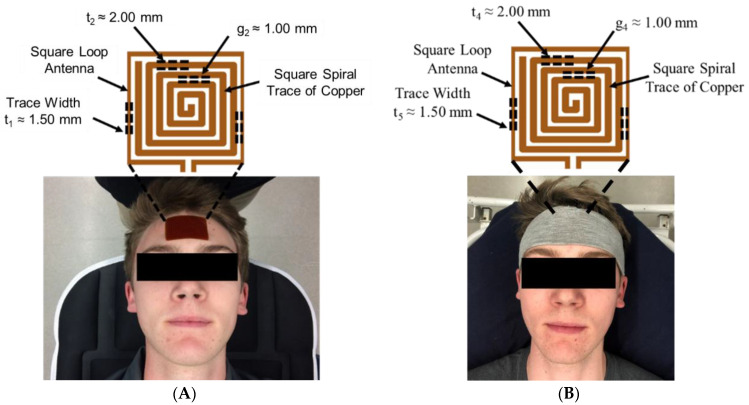
EM skin patch sensor placement (**A**) adhered to forehead (prior work) and (**B**) embedded into a wearable headband (this study). Data was collected from human participants during induce bio-fluid shift (following an IRB approved protocol).

The EM resonant sensor patch was designed and built from a single baseline component: a trace of copper, which was configured into a square planar spiral (Figure 1A), as described in our previous work [18]. Building off previous work, the sensor patch was embedded inside a wearable headband to increase ease of adherence to the forehead (Figure 1B), decrease chances of skin irritation caused by the adhesive, and increase the repeatability of placement of the system during human testing. 

To measure shifts in the resonant frequency response of the rectangular sensor design, a square loop antenna (3.38 cm × 3.38 cm) surrounding the sensor, with a trace width (t_5_) of 1.50 mm, was connected to a coaxial cable using a 50 Ohm SMA (SubMiniature version A) connector. The square loop antenna produced the radiofrequency (RF) wave that interrogated and became electromagnetically coupled with the sensing patch. A vector network analyzer (VNA) (SDR-Kits DG8SAQ, USA) was used to produce an RF sweep in the desired frequency range and measure the return loss S-parameter, or the S_11_ reflection coefficient. Prior to data collection, the VNA was calibrated using the open short match (OSM) method over the desired frequency bandwidth.

### 2.2. Validation of BioFluid Shift

The quantification of the induced biofluid shifts, using techniques involving magnetic resonance imaging (MRI) and intraventricular catheters, would be useful for the validation of this technique; however, these methods require specific surgical expertise and equipment that were not feasible for this study. Instead, the validation of the induced biofluid shifts and the estimates of ICP were performed using optic nerve sheath diameter (ONSD) measurements. We used the same approach to estimate ICP as we previously described in [18]. The relationship between ONSD measurements ICP is described using Equation (1) [18].

(1) ICP=−111.92+(77.36×ONSD)

### 2.3. Data Collection and Analysis for Human Testing–Head-Down Tilt

**Figure 2:**
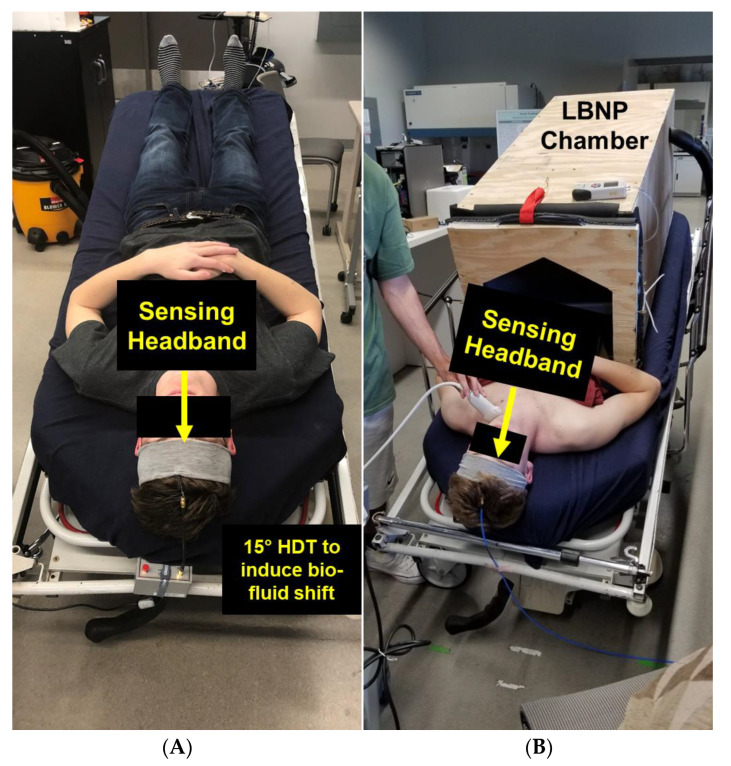
EM skin patch sensor embedded in wearable headband to detect induced bio-fluid shift in (**A**) 15° head down tilt and (**B**) during administration of lower body negative pressure (LBNP).

An emergency department and postanesthesia care unit stretcher (Stryker 1501 PACU Stretcher, Kalamazoo, MI, USA) with tilting capabilities was utilized to induce cephalad biofluid shifts. In this stage of human testing, all participants (*n* = 10) were placed on the stretcher and moved from supine to a 15° head-down tilt, for 30 min, to induce biofluid changes (Figure 2A). This stage of human studies was aimed at simulating the biofluid shift that is experienced by astronauts in a microgravity environment. Data collected during this study included the resonant frequency response of the EM skin patch sensor (every 2.5 min) embedded in the wearable headband, as well as ultrasound images of the optic nerve sheath (every 5 min) for validation. An RF sweep of 10 MHz to 3 GHz was used to find an optimal frequency for obtaining a usable sensor signal response. The VNA was used to interrogate the square sensor with an RF sweep in the range of 250 MHz to 320 MHz, with a bandwidth of 21 MHz, using 201 data points per sweep. The exact frequency range used for each individual was variable, depending on their physiology and the magnitude of the induced biofluid shift. Resonant frequency data were extracted using MatLab 2016a (Natick, MA, USA). The optic nerve sheath diameter was used to estimate ICP to demonstrate the correlation between the shift in ICP and shift in the EM skin patch’s resonant frequency. To monitor participant safety, heart rate and blood pressure were closely monitored throughout this study.

A statistical correlation analysis was performed to determine the relationship between resonant frequency and estimated ICP, obtained using ultrasound. Means and standard deviations were calculated for the estimated ICP (ultrasound) and the sensor’s resonant frequency at each data collection time point. One-way analysis of variance (ANOVA) of the resonant frequency means (calculated at each time point) and a following Bonferroni adjusted multiple comparison test were utilized to determine if there was a statistical difference between the sensor’s resonant frequency responses, corresponding with the shifts in intracranial biofluid volume, over time. A power analysis was performed to determine the statistical power of the system, given α = 0.05, 7 groups, 8 subjects, and an effect size, calculated using the sum of squares between and sum of squares total, from the ANOVA data table. The selected alpha value was obtained to minimize the likelihood of a Type I error. To reduce the effect of Type II errors on this study, a maximum of 4:1 weighting of errors was desired (α = 0.05, and β < 0.20).

### 2.4. Data Collection and Analysis for Human Testing–Lower Body Negative Pressure

In the second stage of human testing, a safe decrease in intracranial biofluids was induced to mimic the physiological response experienced when LBNP countermeasures are administered or when astronauts re-enter a gravitational field. This biofluid shift was induced using a custom-built LBNP chamber (designed and built by the authors).

For the LBNP protocol, 4 participants (n = 4) were recruited and positioned so that the LBNP chamber was sealed at the participant’s iliac crest using a kayak skirt embedded into the lid of the LBNP chamber (Figure 2B). The application of LBNP in a supine position, utilized in this study, induces a shift proportional to the shift in biofluids that pilots experience in high G-force scenarios, or what astronauts experience during re-entry into a gravitational field. The LBNP protocol used for this study started with the application of 0 mmHg, which decreased to −40 mmHg in steps of 10 mmHg every 5 min, then increased back up to 0 mmHg in steps of 10 mmHg every 5 min [27]. Data collected at every pressure over time included heart rate, blood pressure, proposed EM skin patch sensing system’s resonant frequency, and ultrasound images of the optic nerve sheath to estimate changes in ICP.

To evaluate the EM sensing system’s ability to monitor the rapid redistribution of fluid away from the head during the application of LBNP or re-entry into a gravitational field, as a result of orthostatic intolerance, the sensor’s resonant frequency was plotted over time. A statistical correlation analysis was performed between the resonant frequency and estimated ICP. Means and standard deviations were calculated for the estimated ICP and the sensor’s resonant frequency for each pressure. One-way analysis of variance (ANOVA) of the resonant frequency for each of the 9 different pressures, followed by a Bonferroni adjusted multiple comparison, was utilized to determine the sensing system’s ability to detect different intracranial fluid volumes associated with the increasing magnitude of pressure inside the LBNP chamber.

### 2.5. Development of a Predictive Model to Estimate Change in Intracranial Pressure

An important step in validating the use of this sensor technology to monitor aerospace crew health is the ability to output a value or index from the sensor response that relates to usable medical parameters such as ICP. The noninvasive detection of intracranial volumetric changes and estimation of changes in ICP is an integral step toward guiding the administration of countermeasures in astronauts and fighter pilots. The estimation of changes in ICP from the sensor output relies upon the statistical correlation between the change in sensor output and changes in ICP. The primary component of the sensor’s output being analyzed in this study was the resonant frequency; however, the use of other parameters, including phase, impedance, capacitance, and inductance, were also investigated. A simple linear regression model was developed using the data obtained from healthy volunteers in the aforementioned human studies to predict the approximate change in ICP from baseline, based upon the shift in resonant frequency from baseline. The constructed model utilized the change in resonant frequency over time as the predictor variable and the change in ICP values, estimated using ONSD over time, as the response variable. 

Simple linear regression assumes a linear relationship between the predictor and response variables in the form of Equation (2), where *y* is the predicted response, *β*_0_ and *β*_1_ are the optimal coefficients that minimize the sum of squares, and *x* is the predictor variable [28].

(2) $$y=\beta_{0}+\beta_{1}x$$

Analysis of the model was performed using (1) an F-test to determine the significance of regression, (2) the root mean square error (RMSE) to determine the error between the response variable and predicted response, and (3) the coefficient of determination to evaluate the percent of variability of the change in ICP explained by the shift in resonant frequency.

## 3. Results

### 3.1. Validation of BioFluid Shift

During induced biofluid shifts, the shift in intracranial biofluid volume is accompanied by a dilation or constriction of the optic nerve sheath, depending upon if the overall volume is increasing or decreasing. The amount of cerebrospinal fluid in the subarachnoid space surrounding the optic nerve sheath increases as the total intracranial biofluid volume increases. The average diameter increase of the optic nerve sheath during the head-down tilt was approximately 1.32 ± 0.23 mm. Using the diameter of the optic nerve, ICP estimates were obtained, with an average change in ICP of approximately 9.53 ± 1.32 mmHg. Conversely, to validate the induced biofluid shift using the described LBNP protocol, the average ONSD decrease was approximately 1.025 ± 0.4 mm. Using ONSD, the average change in ICP was calculated as approximately −5.83 ± 2.76 mmHg.

### 3.2. Head-Down Tilt Results in Decrease in Resonant Frequency

**Figure 3:**
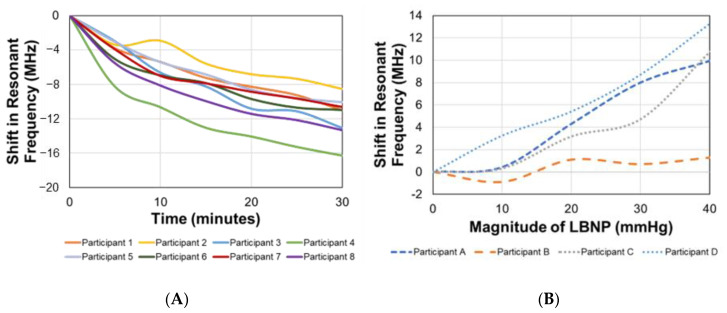
Shifts in EM skin patch sensor’s resonant frequency corresponding with induced bio-fluid shifts over time during (**A**) head down tilt and (**B**) at different pressures during lower body negative pressure protocol.

Over time, the increase in intracranial biofluid volume, induced using a 15° head-down tilt over 30 min, corresponded with a negative shift in the resonant frequency in all participants. Figure 3 represents the shifts in resonant frequency over time. The average shift in resonant frequency from the supine position (time = 0 min) to the end of the 30 min head-down tilt (time = 30 min) was approximately −11.74 ± 2.40 MHz. 

Statistical correlation analysis between the EM skin patch sensor’s resonant frequency and shift in ICP over time yielded R^2^ = 0.89. The linear relationship between these two parameters allows for the utilization of a simple linear regression model. Analysis of the statistical difference between sensor readings at different time points during the head-down tilt was performed. For the seven different time points, the mean and standard deviations of the resonant frequency in MHz, for the eight participants, were 0 ± 0 (0 min), −4.56 ± 1.67 (5 min), −7.01 ± 2.38 (10 min), −9.07 ± 3.08 (15 min), −10.95 ± 3.99 (20 min), −12.23 ± 5.27 (25 min), and −13.76 ± 6.49 (30 min). 

An ANOVA was conducted, followed by a Bonferroni adjusted multiple comparison test, which indicated that the mean shift in resonant frequency in the supine position was significantly different than all other time points during the head-down tilt (Figure 3). Using the sum of squares between and sum of squares total, the effect size (η^2^) was determined to be 0.79. A retrospective power analysis was performed to determine the statistical power of this study (given α = 0.05, 7 groups, 8 subjects, and η^2^ = 0.79). The statistical power for this study was calculated as being greater than 0.99 (β < 0.01). 

### 3.3. Lower Body Negative Pressure Increases Resonant Frequency

As the magnitude of LBNP increased over time, the decrease in intracranial biofluid volume corresponded with a positive shift in the resonant frequency in all participants. Figure 3 represents the shifts in resonant frequency as the magnitude of the pressure increased. The average shift in resonant frequency from baseline (0 mmHg) to the peak pressure (−40 mmHg) was approximately 8.80 ± 5.2097 MHz. 

Statistical correlation analysis between the EM skin patch sensor’s resonant frequency and shift in ICP as LBNP magnitude increased yielded R^2^ = 0.67. The linear relationship between these two parameters allows for the utilization of a simple linear regression model. Analysis of the statistical difference between sensor readings at different pressures during the administration of LBNP was performed. For the six time points at five different pressures, the mean and standard deviations of the resonant frequency in MHz, for the four participants, were 0 ± 0 (0 mmHg at 0 min), −0.51 ± 0.88 (0 mmHg at 5 min), 0.78 ± 1.76 (−10 mmHg at 10 min), 3.50 ± 1.85 (−20 mmHg at 15 min), 5.53 ± 3.66 (−30 mmHg at 20 min), and 8.80 ± 5.2097 (−40 mmHg at 25 min). 

An ANOVA was conducted, followed by a Bonferroni adjusted multiple comparison test, which indicated that the mean shift in frequency at 0 mmHg was significantly different than the mean shifts at −30 mmHg and −40 mmHg (Figure 3). Using the sum of squares between and sum of squares total, the effect size (η^2^) was determined to be 0.65. 

### 3.4. Predictive Model to Estimate Change in Intracranial Pressure

The datasets for the head-down tilt and LBNP studies were combined, using the resonant frequency as the predictor variable and the ICP values, calculated using ultrasound, as the response variable. Using the shifts in resonant frequency corresponding with induced biofluid shifts (correlated with shifts in ICP), a predictive simple linear regression model was built using Equation (3).

(3) y=−0.7199x+0.8899

**Figure 4:**
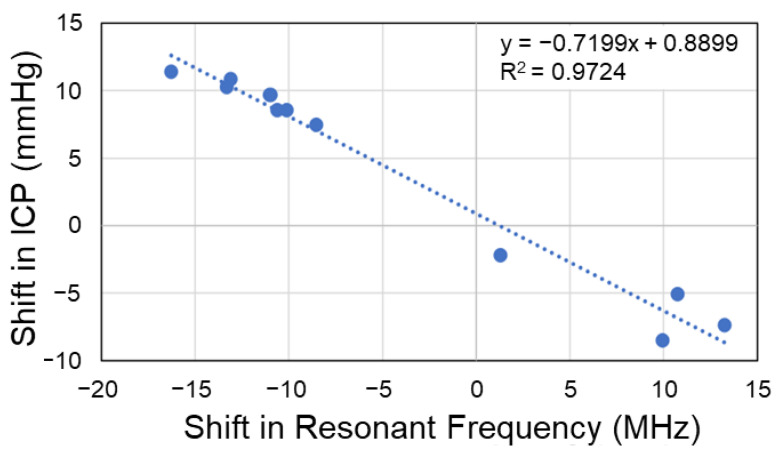
Simple linear regression model relating shifts in EM skin patch sensor’s resonant frequency (predictor) with intracranial pressure (response). RMSE = 1.24 mmHg.

Using the R output summary of the linear model, analyses of the performance and adequacy of the model were performed. The developed model (R^2^ = 0.97) explains 97% of the variance in ICP during induced biofluid shifts, using the shift in resonant frequency (Figure 4). A global F-test was performed to determine the significance of regression, yielding an F-statistic of 352.5 at 1 and 10 degrees of freedom, with a *p*-value of 3.98 × 10^−9^. A p-value less than 0.05 indicated adequacy of the created model. To analyze accuracy of the estimations, root mean square error (RMSE) calculations were performed, with RMSE = 1.24.

## 4. Discussion

In this study, we demonstrated the ability of an EM skin patch sensor embedded in a wearable headband to detect induced shifts in intracranial biofluid volume. Electromagnetic waves penetrate through biological tissues, such as bone, more effectively than their mechanical counterparts, providing a larger range of clinical applications. Additionally, the SAR values obtained in this study (less than 0.16 W/kg averaged over a 1 g cube) were more than an order of magnitude below the maximum allowed SAR (1.6 W/kg over a 1 g cube). 

The ability of the sensor to detect both increases and decreases in intracranial biofluid volume, as well as its correlation with shifts in ICP, is a promising step towards the noninvasive monitoring of ICP shifts in an aerospace environment. The proposed sensing system presents the possibility of directly tracking volumetric increases and decreases inside the cranial cavities of astronauts and pilots, instead of relying upon secondary physiological indicators. Additionally, this system addresses three major limitations regarding intracranial pressure/volume monitoring: invasiveness, penetration depth, and the need for specialized medical imaging equipment. Through the simplicity of the application and operation of our EM skin patch sensor, several risks and limitations associated with current methods for measuring intracranial volume/pressure could be significantly reduced while opening up new possibilities for clinical and aerospace medicine applications.

The results of this study indicate that shifts in resonant frequency can be leveraged to predict shifts in ICP over time. This pressure monitoring capability may be utilized to guide the administration of the Valsalva maneuver or anti-G suit in fighter pilots, or LBNP and other countermeasures in astronauts in a microgravity environment.

### Potential Applications

This sensor technology may be harnessed as a point-of-care biomedical diagnostic tool to track the progression of biofluid volume irregularities in the cranial cavity in a variety of settings, including aerospace environments. A long-term goal of this research is to develop a wearable body sensor network to monitor intracranial biofluid shifts associated with an aerospace environment and to provide easy-to-interpret medical updates in the form of alert systems and countermeasure recommendations. 

In regards to biofluid shifts in fighter pilots, a primary concern is G-force-induced loss of consciousness (G-LOC). Exposure to high G-force accelerations can result in alterations in blood pressure and the redistribution of blood throughout the body. The redistribution of blood volume towards the lower extremities, as a result of the increased hydrostatic pressure gradient, is associated with nausea, vomiting, visual disturbances, and headaches as pilots approach intracranial hypotension. Anti-G suits and the Valsalva maneuver are in place to maintain sufficient cerebral blood volume and avoid loss of consciousness. The real-time monitoring of intracranial fluid volume would provide more information to guide and optimize the administration of these countermeasures through personalized recommendations. One approach to avoid loss of consciousness is subjecting pilots to high G-forces during training, in a centrifuge, and determining their tolerance. Then, as the G-forces increase, as monitored inside the aircraft, countermeasures are put in place to avoid reaching their max tolerance.

Orthostatic intolerance is a serious concern regarding fighter pilots in a high G-force environment and astronaut safety during long-duration spaceflight missions, and is a direct effect of cardiovascular deconditioning [29]. During orthostatic intolerance, the cardiovascular system is no longer able to properly autoregulate, and significant biofluid shifts can occur as their exposure to G-forces suddenly increases, such as during complex aerial maneuvers or re-entry into a planetary gravitational field. This can result in the rapid shift and pooling of fluid in the lower extremities, away from the brain, which involves the risk of the individual losing consciousness [29]. Current physiological monitoring to track the risk of G-force-induced loss of consciousness relies upon heart rate monitoring, electroencephalography, or electromyography of the gastrocnemius muscles [30,31,32]. These techniques measure a secondary physiological change instead of directly obtaining information regarding the shift in cerebral blood volume, which is the primary mechanism believed to cause G-force-induced loss of consciousness. The limitation of these methods is that other physiological adaptations can lead to alterations in gastrocnemius muscle activation or heart rate, which may not always be indicative of fluid shift and impending loss of consciousness, thus highlighting a need for a noninvasive monitoring tool to track pilot health and guide countermeasures, relevant to cerebral blood volume, aimed at preventing loss of consciousness. The proposed EM skin patch sensing system aims at addressing these limitations to track decreases in cerebral blood volume instead of relying upon secondary physiological indicators. 

Systems integration of the designed EM skin patch sensor with our other work aims to develop a portable data acquisition system [33], and predictive algorithms aim at providing a viable alternative to the monitoring and medical assessment of biofluid shifts induced in a microgravity environment. Based on the estimated increases in ICP, countermeasures can be recommended. During the administration of the countermeasure, continuous monitoring of the intracranial biofluids could provide important, individualized details regarding the appropriate duration for each countermeasure.

The primary limitation of this work is sample size. This work demonstrates a relationship between shifts in resonant frequency and shifts in ICP. However, additional studies will be needed to robustly characterize this relationship. The physics of the sensor dictates that the resonant frequency shift is in response to a volumetric shift, not a pressure shift. Therefore, in different subjects, the pressure associated with a specific volumetric shift may vary. As a result, a diverse range of subject sizes—male and female—should be selected in future studies to fully characterize intersubject effects on predicting the shift in ICP based on the resonant frequency shift.

## 5. Conclusions

The EM skin patch embedded into a wearable headband that was examined in this study was able to detect induced intracranial biofluid volume changes simulating those seen in an aerospace environment. This detection capability relies upon the sensor’s ability to detect alterations in the effective electric permittivity of a layered system. The results demonstrate an ability for the sensor to detect an increase in intracranial fluid and to estimate the shift in ICP. This is an integral step towards creating an approach to noninvasively monitor shifts in ICP. This work, combined with the optimization of the wearable form factor and incorporation of a portable data acquisition system, may lead to the development of a wearable body sensor network to monitor intracranial biofluid shifts and provide easy-to-interpret medical updates in the form of alert systems and countermeasure recommendations for pilots and astronauts in an aerospace environment.

## Data Availability

The data presented in this study are available on request from the corresponding author.
